# Design of a *Helicobacter pylori* multi-epitope vaccine based on immunoinformatics

**DOI:** 10.3389/fimmu.2024.1432968

**Published:** 2024-08-23

**Authors:** Man Cui, Xiaohui Ji, Fengtao Guan, Guimin Su, Lin Du

**Affiliations:** ^1^ Research and Development Centre, Beijing Zhifei Lvzhu Biopharmaceutical Co., Ltd., Beijing, China; ^2^ Beijing Bacterial Vaccine Engineering Research Centre, Beijing, China

**Keywords:** *Helicobacter pylori*, immunoinformatics, multi-epitope vaccine, vaccine, multiepitope based vaccine

## Abstract

*Helicobacter pylori* (*H. pylori*) is an infectious bacterium that colonizes the stomach of approximately half of the global population. It has been classified as a Group I carcinogen by the World Health Organization due to its strong association with an increased incidence of gastric cancer and exacerbation of stomach diseases. The primary treatment for *H. pylori* infection currently involves triple or quadruple therapy, primarily consisting of antibiotics and proton pump inhibitors. However, the increasing prevalence of antibiotic resistance poses significant challenges to this approach, underscoring the urgent need for an effective vaccine. In this study, a novel multi-epitope *H. pylori* vaccine was designed using immunoinformatics. The vaccine contains epitopes derived from nine essential proteins. Software tools and online servers were utilized to predict, evaluate, and analyze the physiochemical properties, secondary and tertiary structures, and immunogenicity of the candidate vaccine. These comprehensive assessments ultimately led to the formulation of an optimal design scheme for the vaccine. Through constructing a novel multi-epitope vaccine based on immunoinformatics, this study offers promising prospects and great potential for the prevention of *H. pylori* infection. This study also provides a reference strategy to develop multi-epitope vaccines for other pathogens.

## Introduction

*Helicobacter pylori* (*H. pylori*) is a microaerophilic, Gram-negative bacterium that colonizes the mucous layer of the human gastric epithelium (1). Infection with *H. pylori* typically occurs during childhood and is primarily transmitted among individuals through fecal-oral and oral-oral routes (2). Approximately half of the global population is infected with *H. pylori*, with infection rates varying across countries and regions. In western countries, the infection rate ranges from 20% to 40%, while in Asia and developing countries, it can reach as high as 70% to 90% (3, 4). *H. pylori* infection has been associated with various gastrointestinal diseases in humans, including chronic gastritis, gastric ulcers, mucosa-associated lymphoid tissue (MALT) lymphomas, and gastric cancer. The inflammation and damage caused by *H. pylori* infection are responsible for approximately 75% of stomach cancers and 5.5% of malignancies worldwide. Therefore, the World Health Organization classifies H. pylori as a Group I carcinogen (5–7).

The current therapeutic strategies for *H. pylori* infection primarily involve triple or quadruple therapy, which encompasses a combination of two or three antibiotics, proton pump inhibitors (PPIs), and bismuth salts (8). Nevertheless, it has been documented that *H. pylori* is progressively developing resistance to conventionally administered antibiotics, culminating in a deterioration of the efficacy of antimicrobial regimens. Furthermore, antimicrobial therapy is beset with several disadvantages, such as high costs, severe adverse effects, and the looming possibility of reinfection (9). Consequently, there exists an urgent imperative to explore more efficacious methodologies for the management of *H. pylori* infection.

Vaccination has been conclusively validated as an efficacious approach for the prevention and treatment of infectious diseases. Extensive research efforts have been dedicated to the development of a *H. pylori* vaccine. However, the development of a mature *H. pylori* vaccine that provides satisfactory immune protection continues to pose significant challenges (10–12). The efficacy of monovalent vaccines, which are composed of a single *H. pylori* antigen, is limited. In contrast, multivalent vaccines that target multiple *H. pylori* antigens are anticipated to exhibit superior immunogenicity compared to monovalent vaccines (13–15). Nevertheless, the construction and expression of recombinant subunit vaccines containing several antigens are complicated by the large molecular weights of the individual protein antigens from *H. pylori*. Epitopes are specific regions on an antigen molecule that are specifically recognized by antibodies or T cell receptors. Vaccines designed based on epitopes represent an innovative direction in vaccine development and offer an effective strategy for the development of multivalent *H. pylori* vaccines (16, 17).

Immunoinformatics has emerged as a pioneering field in the investigation of novel vaccines. With the rapid evolution of genomics, proteomics, human immunology, and structural biology, the employment of immunoinformatics tools to predict and identify neoantigens and epitopes has revolutionized the approach to pathogenic vaccine development. The synergistic integration of reverse vaccinology and immunoinformatics in the design of multi-epitope vaccines represents a promising avenue for future vaccinological endeavors.

*H. pylori* infection in the host involves the coordinated action of numerous bacterial proteins. These include flagellar proteins, which facilitate motility and aid in traversing the viscous stomach environment (18). Additionally, *H. pylori* produces, an enzyme that catalyzes the conversion of urea into carbon dioxide and ammonia, thereby increasing the local pH and creating a less acidic microenvironment around the bacterium. This allows it to survive in the otherwise harsh acidic conditions of the stomach. The active center of urease is located on the urease B subunit (UreB) (19, 20). Adhesion proteins mediate binding to the gastric epithelial cells, promoting colonization (21). Key adhesion proteins in *H. pylori* include Blood group antigen-binding adhesin A (BabA), Sialic acid binding adhesin (SabA), Adhesion associated lipoproteins A/B (AlpA/AlpB), among others (22, 23). Furthermore, *H. pylori* possesses various virulence factors that lead to alterations in cell signaling, cytoskeletal rearrangements, and induction of pro-inflammatory responses (24, 25). Cytotoxin-associated antigen L (CagL) and cytotoxin-associated antigen A (CagA) are part of the type IV secretion system (T4SS), which is encoded by Cag Pathogenicity Island (Cag PAI). CagA is transported into host cells to exerts its virulent effects, a process in which CagL is involved (26–28). Vacuolar cytotoxin A (VacA) serves as a principal virulence factor, capable of inducing cellular vacuolization (29). Neutrophil activating protein (NAP) is released by *H. pylori* bacteria near the monolayer region of the gastric epithelium, and triggers the inflammatory response (30). Gamma-glutamyl transpeptidase (GGT) facilitates the conversion of glutamine to glutamic acid and ammonia, as well as catalyzes the transformation of glutathione into glutamic acid and cysteine. GGT can induce cell cycle arrest, apoptosis, and necrosis of gastric epithelial cells (31).. As a crucial virulence factor, High-temperature requirement A (HtrA) functions as both a molecular chaperone and serine protease, playing a significant role in bacterial stress response and the cleavage of the human cell adhesion molecules (32–34).

In this study, a comprehensive literature review and analysis were conducted to identify candidate proteins of *H. pylori* with potential for vaccine development. Considering the important role of these proteins in *H. pylori* infection, UreB, SabA, BabA, VacA, CagA, GGT, HtrA, NAP and CagL were selected as antigens to design this vaccine. Online servers ABCpred and IEDB were utilized to predict B cell and T cell epitopes from these nine candidate proteins. The VaxiJen application predicted antigenicity. Dominant epitopes were selected based on antigenicity prediction results, and linked by different linkers. A complete multi-epitope antigen sequence was designed and subjected to rigorous evaluation for the rationality of vaccine design through analysis of physicochemical properties (ExPASy ProtParam), secondary (Prabi server) and tertiary structures (I-TASSER), molecular docking (Cluspro2.0), and dynamic simulation (Gromacs-2023). The novel immunogenicity of the multi-epitope vaccine was also simulated using an online server. The codon-optimized DNA sequence of the vaccine was subsequently cloned *in silico* into a protein expression vector, paving the way for subsequent experimental validation.

## Methods

### Selection of target proteins

**Figure 1:**
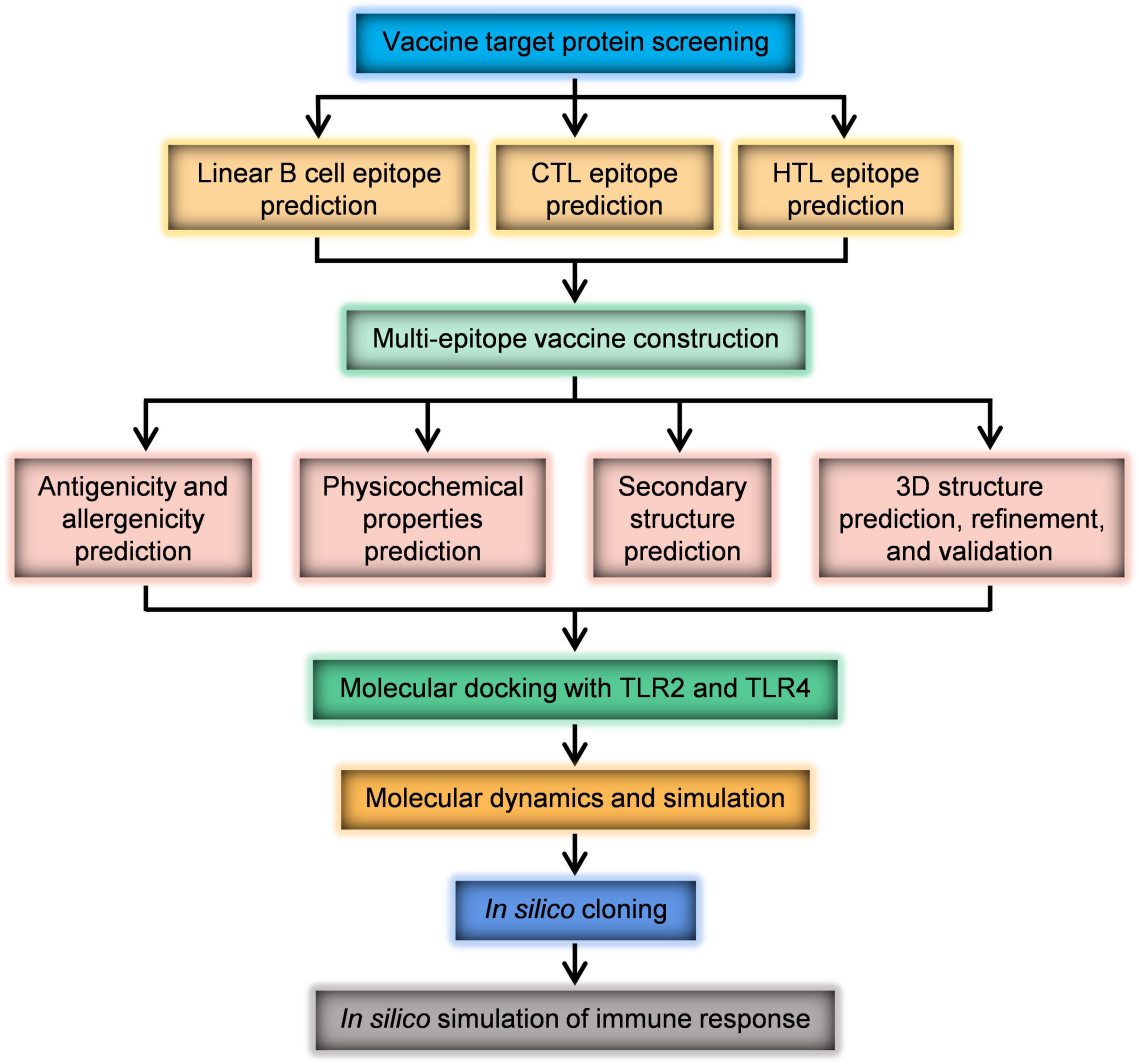
The process of designing the multi-epitope vaccine.

In this study, the selection of source proteins for a multi-epitope vaccine was based on their virulence, importance, antigenicity, and immunogenicity. The proteins chosen were UreB, SabA, BabA, VacA, CagA, GGT, HtrA, NAP, and CagL. The protein information of *H. pylori* strain 26695 was obtained from the UniProt (35) (https://www.uniprot.org/), a universal protein resource database. Protein sequences were downloaded in FASTA format. The research process is illustrated in **Figure 1**.

### B cell and T cell epitope prediction

The online prediction tool ABCpred achieved 72.94% accuracy in predicting linear B-cell epitopes based on amino acid anchoring pairs (APC) (36). The ABCpred (37) (https://webs.iiitd.edu.in/raghava/abcpred/ABC_submission.html) and immune epitope database IEDB (38, 39) (http://tools.iedb.org/main/bcell/) were utilized to predict linear B lymphocyte epitopes of candidate proteins. Additionally, the online tools available on IEDB website (40, 41) (http://tools.iedb.org/main/tcell/) were employed to predict T cell epitopes. According to the epitope prediction results, the dominant epitopes of each protein with higher prediction rate and score were selected.

### Multi-epitope vaccine construction

Linkers between epitopes can offer the amino acid residue the greatest degree of flexibility and prevent the expected epitopes from folding (42). Dominant linear B-cell, cytotoxic T Lymphocytes (CTL), and helper T Lymphocytes (HTL) epitopes from nine target proteins were sequentially linked using glycine-proline-glycine-proline-glycine (GPGPG), lysine-lysine (KK) and alanine-alanine-tyrosine (AAY), respectively, to generate multi-epitope vaccines.

### Antigenicity and allergenicity prediction

The VaxiJen application (http://www.ddg-pharmfac.net/vaxijen/VaxiJen/VaxiJen.html) is a sequence alignment method-independent tool that utilizes hydrophobic amino acids, molecular weight, and polarity as antigen characteristics. The partial least squares algorithm is employed to establish a model for predicting protein antigenicity. VaxiJen demonstrates a prediction accuracy of 70-89%, with a threshold of 0.4 considered indicative of antigenicity (43). The AllerTOP server (http://www.ddg-pharmfac.net/AllerTOP/index.html) is a predictive tool that has been trained on a database of allergens and non-allergens. It predicts the allergenicity of proteins based on their primary physical and chemical properties achieving an accuracy of approximately 94%. Upon submission to the AllerTOP server, a protein will be predicted as “Probable Allergen” or “Probable Non-allergen” (44).

### Physicochemical properties evaluation

ExPASy ProtParam (45) (http://web.expasy.org/protparam/) was used to determine the physicochemical parameters of vaccine constructs based on the sequence and pKa values of amino acids contained within the protein (46). The parameters computed by ProtParam include the molecular weight (MW), theoretical isoelectric point (pI), amino acid composition, estimated half-life, instability index (considered stable if <40), aliphatic index and grand average of hydropathicity (GRAVY). ProtParam utilizes the “N-end rule,” which associates the half-life of a protein with the properties of its N-terminal residue (47, 48). The aliphatic index of a protein is calculated based on the volume occupied by aliphatic side chains, which contributes positively to the thermal stability of globular proteins (49). The GRAVY value is calculated by dividing the total hydropathy of all amino acids by the total amount of amino acids in the protein, indicating the hydrophobic or hydrophilic nature of the protein (50).

### Secondary structure prediction

The secondary structure of vaccines was predicted using the online tool Prabi server (https://npsa-prabi.ibcp.fr/cgi-bin/npsa_automat.pl?page=npsa_sopma.html) (51). The number of conformational states was set to 4 (Helix, Sheet, Turn, Coil), while other options remain at their default settings.

### Tertiary structure prediction, refinement, and validation

The tertiary structure of vaccines was generated using the online server I-TASSER (https://zhanglab.ccmb.med.umich.edu/I-TASSER/) based on amino acid sequences (52). According to the C-scores of structures, the best structure was chosen for further refinement. The side chains of amino acids was repacked to optimize the quality and stability model structures by using the online tool GalaxyRefine (53) (https://galaxy.seoklab.org/). After structure optimization, the optimal tertiary model structure of the vaccine was verified using the PROCHECK module in SAVES v6.0 (https://saves.mbi.ucla.edu/) and the result was shown in the Raman diagram. ProSA–web (54) (https://prosa.services.came.sbg.ac.at/prosa.php) was also employed to obtain the Z score, a parameter representing the rationality of the tertiary model structure.

### Molecular docking and dynamic simulation

Cluspro2.0 (55) (http://cluspro.bu.edu/login.php) is an automatic and efficient rigid-body protein docking server that is capable of predicting protein-protein interactions. The best optimized tertiary model structure of the multi-epitope vaccine was chosen. The molecular dockings between the vaccine and Toll-like receptor 2 (TLR2, PDB ID 3A7C) or Toll-like receptor 4 (TLR4, PDB ID 2Z63) were performed by Cluspro2.0 server with all parameters set to their default values. The docked structures were visualized by PyMol (56). The stability of vaccine-receptor complexes was evaluated utilizing the iMODS server (https://imods.iqf.csic.es/) (57, 58). Molecular dynamics simulation refers to a collection of molecular simulation methods that use Newtonian mechanics to simulate the movement of molecular systems. To analyze molecular motion and assess the stability of the docking complex, molecular dynamics simulations were conducted using Gromacs-2023 software (59). The docking complex was solvated in a cubic box using spc216 water solvent. Subsequently, CL^-^ ions were added to neutralize the charged protein complex, initial energy minimization included 50,000 steps of the steepest descent method. Equilibration was done in phases, and production simulations ran for 100 ns using NVT and NPT ensembles. Temperature was set at 300 K, and pressure was maintained at 1 atm. Then a 100 ns molecular dynamics (MD) simulation was conducted. The entire MD simulation utilized the all-atomic OPLS force field. The analysis was conducted using GROMACS tools, and the graphs were created with Origin 2021.

### Immune simulation

*In silico* immune simulations were carried out using C-ImmSim online server (https://kraken.iac.rm.cnr.it/C-IMMSIM/index.php). C-IMMSIM is derived from a universal simulation platform that appropriately describes the role of immune responses in different human pathologies (60). Three injections, each containing with 1000 vaccine proteins, were administrated one month apart at 1, 90, and 270 time-steps (every three steps represent one day in real life) with total 540 simulation steps. All other simulation parameters were kept at their default settings.

### *In silico* cloning

The amino acid sequence of the multi-epitope vaccine was submitted to the website server (https://www.novopro.cn/tools/codon-optimization.html) for codon optimization. The coding sequence of the vaccine was then cloned into the pET-28a(+) vector using SnapGene software.

## Results

### Prediction of epitopes

To improve the prediction accuracy, two independent methods, ABCpred and IEDB were utilized to predict linear B-cell epitopes. For the ABCpred prediction results, we use the score as the selection criterion, for the IEDB prediction results, we prioritize based on ranking. We give preference to peptides that have a higher score in the ABCpred results and are top-ranked in the IEDB predictions, especially when there is an overlap between the two. To ensure antigenicity, the VaxiJen application is used to predict the antigenicity of the selected peptides. Peptides scoring above the threshold of 0.4 were selected to be the dominant linear B-cell (LBL) epitopes (**Table 1**). CTL epitopes were predicted using the Major Histocompatibility Complex I (MHC I) binding prediction tool of IEDB, and epitopes with high scores and antigenicity were selected for multi-epitope vaccine construction (**Table 2**). The MHC II binding prediction tool of IEDB was used to predict HTL epitopes, and the sequences with higher percentile ranks and high antigenicity were selected as the dominant HTL epitopes (**Table 3**).

**Table 1:** The scores and antigenicity of predicted linear B-cell epitopes.

Name	Start site	Sequence	Length (aa)	Score	Antigenicity
UreB	154	TTMIGGGTGPADGTNA	16	0.92	1.0996
BabA	204	TYTYTCSGQGNNNCSP	16	0.94	1.218
SabA	49	KELNDKYEQLNQYLNQVA	18	0.87	0.4238
VacA	400	NADGTIKVGGYKASLTTNA	20	0.95	1.1731
CagA	3	NETIDQTRTPDQTQSQ	16	0.96	0.991
HtrA	4	LKTIRIYSYHDSIKDS	16	0.93	0.5182
GGT	349	AKKIFDTIQPDTVTPS	16	0.91	0.5432
CagL	71	AAIALRGDLALLKANFEA	18	0.9	0.7731
NAP	15	IVLFMKVHNFHWNVKGTD	16	0.9	1.456

**Table 2:** The scores and antigenicity of predicted CTL epitopes.

Name	Location	Sequence	Allele	Length (aa)	Score	Antigenicity
UreB	467-475	IPTPQPVYY	HLA-B*35:01	9	0.995265	0.7004
BabA	715-723	AELKYRRLY	HLA-B*44:03	9	0.985894	1.3304
SabA	5-13	FLLSLSLSL	HLA-A*02:01	9	0.920702	1.7958
VacA	307-316	KTHIGTLDLW	HLA-B*57:01	10	0.991128	0.5692
CagA	601-609	AEAKSTGNY	HLA-B*44:03	9	0.937662	1.143
HtrA	370-378	RLSDDVQGV	HLA-A*02:03	9	0.963168	1.2643
GGT	498-507	VSAPRFHMQW	HLA-B*57:01	10	0.995623	1.6739
CagL	104-113	MSSPELLLTY	HLA-B*57:01	10	0.906037	0.6337
NAP	81-89	ETKTSFHSK	HLA-A*68:01	9	0.9477	0.9477

**Table 3:** The percentile ranks and antigenicity of predicted HTL epitopes.

Name	Location	Sequence	Allele	Percentile rank	Antigenicity
UreB	406-420	LSKYTINPAIAHGIS	HLA-DRB3*02:02	0.01	0.6540
BabA	666-680	ANFQFLFNMGVRMNL	HLA-DRB3*02:02	0.01	1.6171
SabA	1-15	MKKRFLLSLSLSLSL	HLA-DRB3*02:02	0.71	0.9369
VacA	1169-1183	GSTNFKSNSNQVALK	HLA-DRB3*02:02	0.01	1.3185
CagA	178-192	GNQIRTDQKFMGVFD	HLA-DRB1*03:01	0.12	0.5419
HtrA	74-88	SKDGYIVTNNHVIDG	HLA-DRB3*02:02	0.8	0.7683
GGT	486-500	NVIDYNMNISEAVSA	HLA-DRB3*02:02	0.11	0.8437
CagL	159-173	SLKAYQSNIGGTASL	HLA-DRB3*02:02	0.99	1.1898
NAP	15-29	IVLFMKVHNFHWNVK	HLA-DRB3*02:02	9.8	1.2673

### Design and construction of multi-epitope vaccine

**Figure 2:**
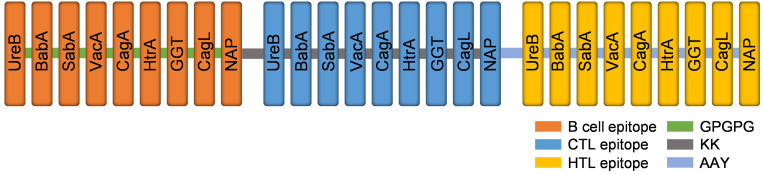
The structure diagram of the multi-epitope vaccine. Linear B cell epitopes (orange), CTL epitopes (blue) and HTL epitopes (yellow) of nine target proteins were fused by GPGPG (green), KK (grey) and AAY (light blue) linkers.

The predicted epitopes of the 9 candidate proteins were tandemly connected in the order of B cell epitope, CTL epitope, and HTL epitope. The order of epitopes was adjusted and the antigenicity, allergenicity, and physicochemical properties were predicted. The arrangement order that met all standards was selected to construct the multi-epitope vaccine. Adding linkers between epitopes not only effectively prevents the formation of new epitopes, but also promotes epitope presentation (61, 62). In this research, the LBL epitopes were linked by linker GPGPG, the CTL epitopes were connected by linker AAY which is a preferential cleavage site for proteasome, and HTL epitopes were connected by KK which is a target cleavage site for lysosomal protease (63, 64). The construction of the multi-epitope vaccine is shown in **Figure 2**.

### Prediction of vaccine features

The multi-epitope vaccine designed in this study comprises 458 amino acid residues, with a computed molecular weight of 49.47 kDa and a theoretical isoelectric point (PI) of 9.7, which imply the fundamental properties of the protein. The predicted antigenicity probability of the vaccine is 0.9674, surpassing the threshold value of 0.4, indicating a high likelihood of eliciting an immune response. Additionally, the vaccine is predicted to be non-allergenic. The instability index is predicted to be 15.98, suggesting the construct is stable. The aliphatic index is predicted to be 71.44, indicating that the construct possesses thermostability. The GRAVY score is predicted to be -0.423, indicating the hydrophilic character of the vaccine, which enhances its interaction with other proteins. Furthermore, the vaccine design is predicted to be soluble in an aqueous environment, with a solubility score of 0.498. The computed half-life of the vaccine is 7.2 hours in mammalian reticulocytes, greater than 20 hours in yeast, and greater than 10 hours in *Escherichia coli* (**Table 4**).

**Table 4:** Evaluation of the vaccine construct’s antigenicity, allergenicity, and physicochemical properties.

Features	Assessment
Amino acid number (aa)	458
Molecular weight	49.47
Theoretical isoelectric point (pI)	9.70
Antigenicity	0.9674
Allergenicity	non-allergen
Solubility	0.498 (soluble)
Instability index	15.98 (stable)
Aliphatic index	71.44
Grand average of hydropathicity (GRAVY)	-0.423
Estimated half-life	7.2 h (mammalian reticulocytes, *in vitro*) >20 h (yeast, *in vivo*) >10 h (*Escherichia coli*, *in vivo*)

### Prediction of secondary structure

**Figure 3:**
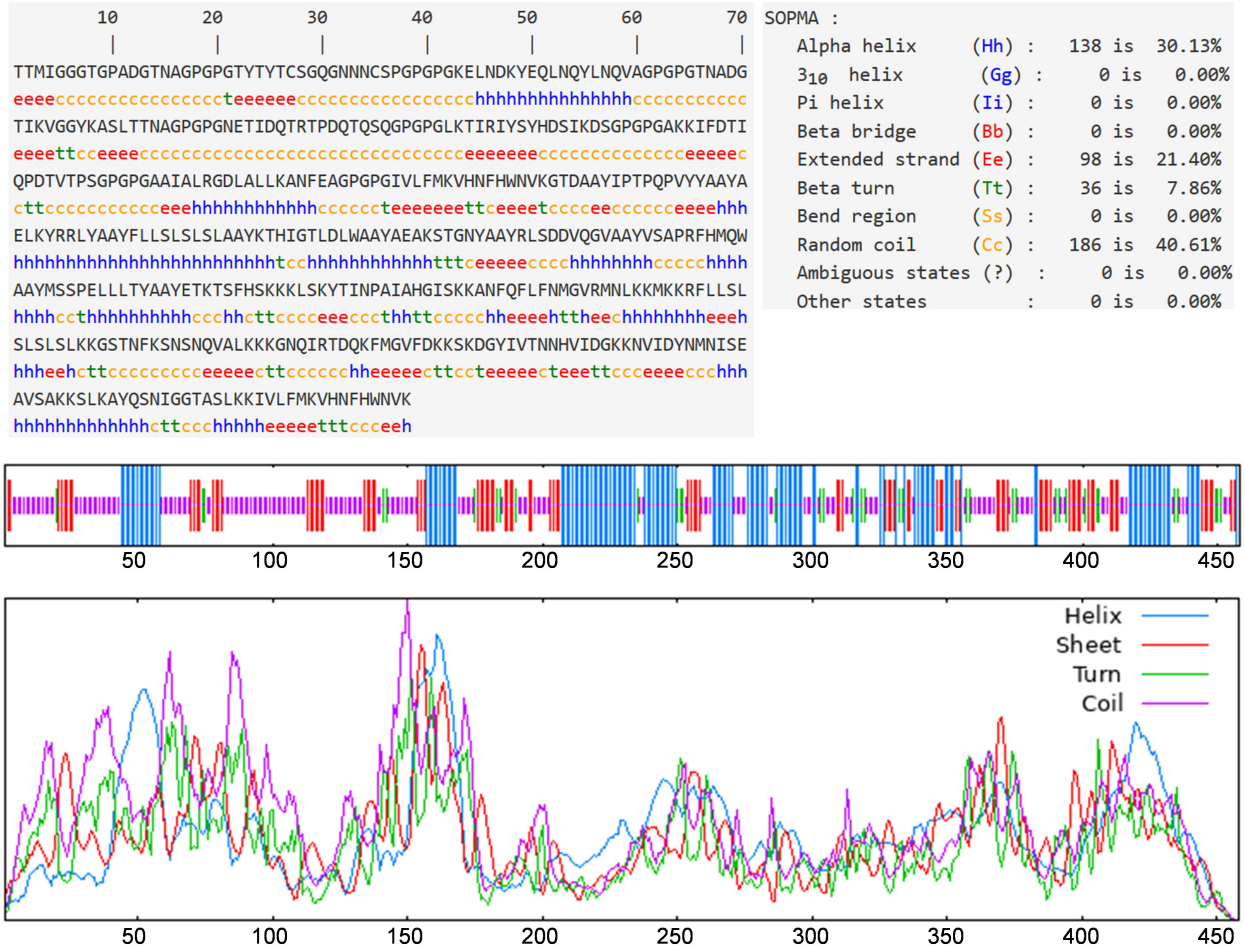
Prediction results of secondary structure. Alpha helix, extended strand, beta turn, and random coil accounted for 30.13%, 21.40%, 7.86%, and 40.61%, respectively.

The bioinformatics tool Prabi was employed to evaluate the secondary structure and to enumerate the number of amino acids in each conformational state. The respective proportions of alpha helices, extended strands, beta turns, and random coils were calculated to be 30.13%, 21.40%, 7.86%, and 40.61% (as depicted in **Figure 3**). Notably, the highest proportion was observed for random coils, suggesting a considerable flexibility within the vaccine structure. The presence of beta turns implies that the vaccine is likely to be easily recognized by antibodies.

### Tertiary structure modeling, refinement and validation

**Figure 4:**
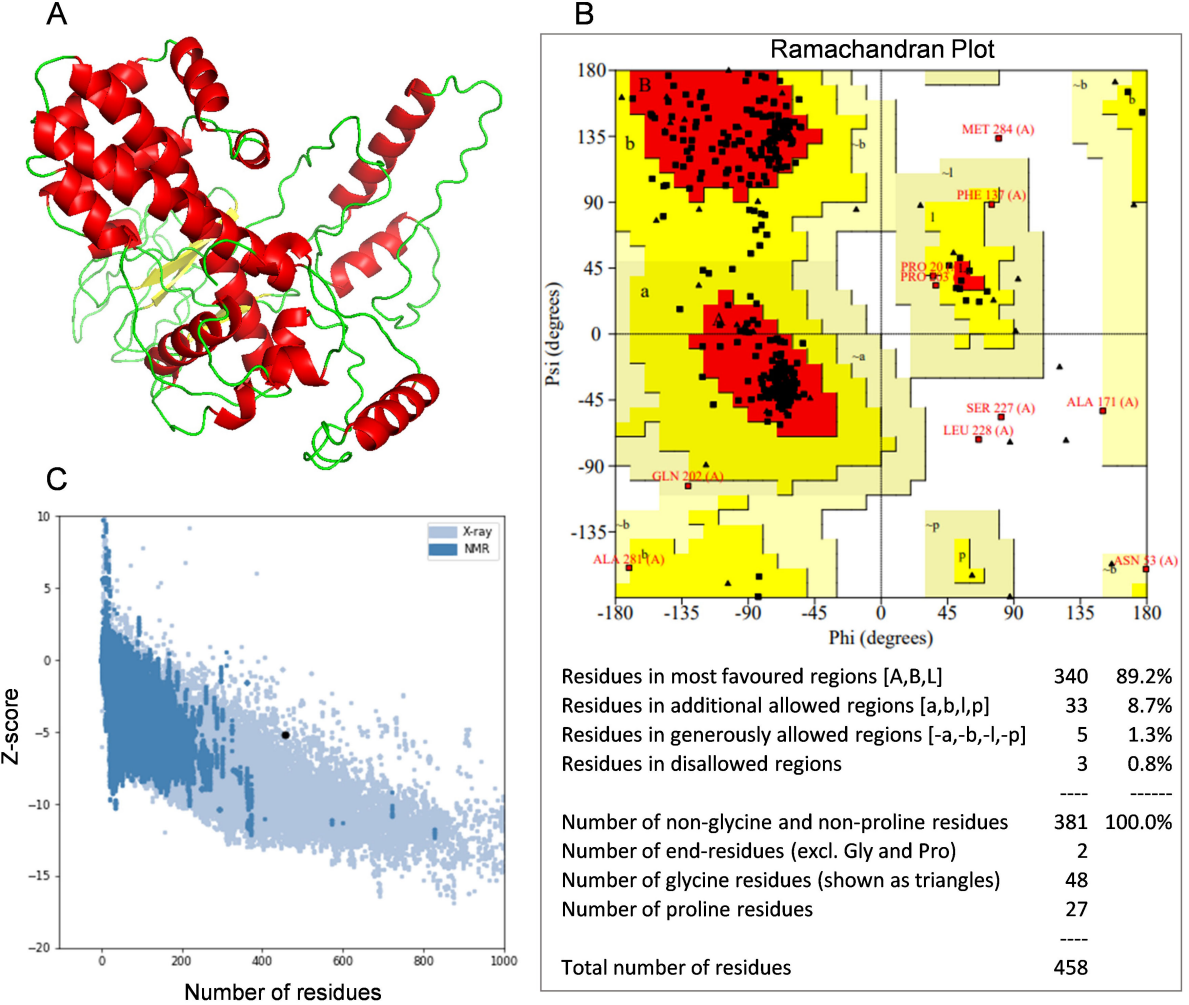
Tertiary structure model and model validation. **(A)** The refined tertiary structure model (red color depicts α-helix, green color depicts coiled structure and yellow color depicts β-strand). **(B)** Ramachandran plot of refined tertiary structure model (89.2% of amino acid residues were in most favored regions). **(C)** A ProSA validation of refined tertiary structure model by Z-score.

The I-TASSER online platform was utilized to predict the tertiary structure of the multi-epitope vaccine. The model with the highest confidence score was selected for further refinement, with secondary structures annotated in distinct colors (**Figure 4A**). The Ramachandran plot analysis revealed that 89.2% of the amino acid residues fell within the most favored regions, 8.7% within the additionally allowed regions, 1.3% within the generously allowed regions, and 0.8% within the disallowed regions, thereby corroborating the reliability of the constructed tertiary structure model (**Figure 4B**). The z-score is an indicator of the overall model quality, quantifying the deviation of the structure’s total energy from the energy distribution expected for random conformations (65). The ProSA analysis yielded a z-score of -5.15 for the multi-epitope vaccine, suggesting that the optimized tertiary structure model possesses good quality (**Figure 4C**).

### Molecular docking and dynamics simulation of vaccine-TLR complex

**Figure 5:**
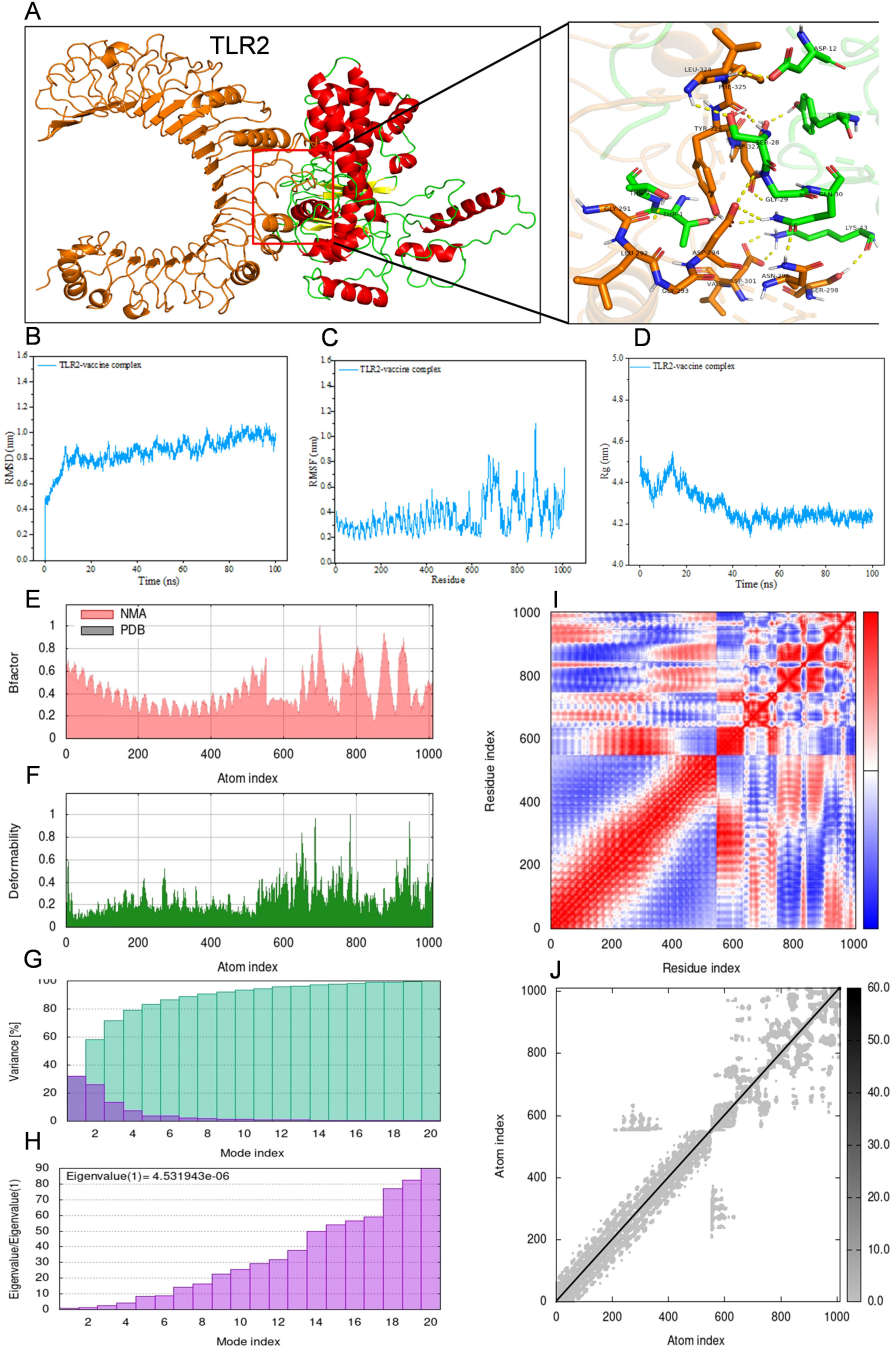
Molecular docking between vaccine and human TLR2 and iMODS results of the docking complex. **(A)** Molecular docking result of vaccine with TLR2. The residues involved in the hydrogen bond are shown on the right-hand side. **(B–D)** Molecular dynamic simulation of the vaccine-TLR2 complex, including RMSD value of the complex backbone, RMSF value of side-chain residues, and radius of gyration during the molecular dynamic simulation. **(E–J)** Results of iMODS of vaccine-TLR2 docking complex. **(E)** B-factor; **(F)** Deformability plot; **(G)** Variance; **(H)** Eigenvalue; **(I)** Covariance matrix analysis; **(J)** Elastic network model.

**Figure 6:**
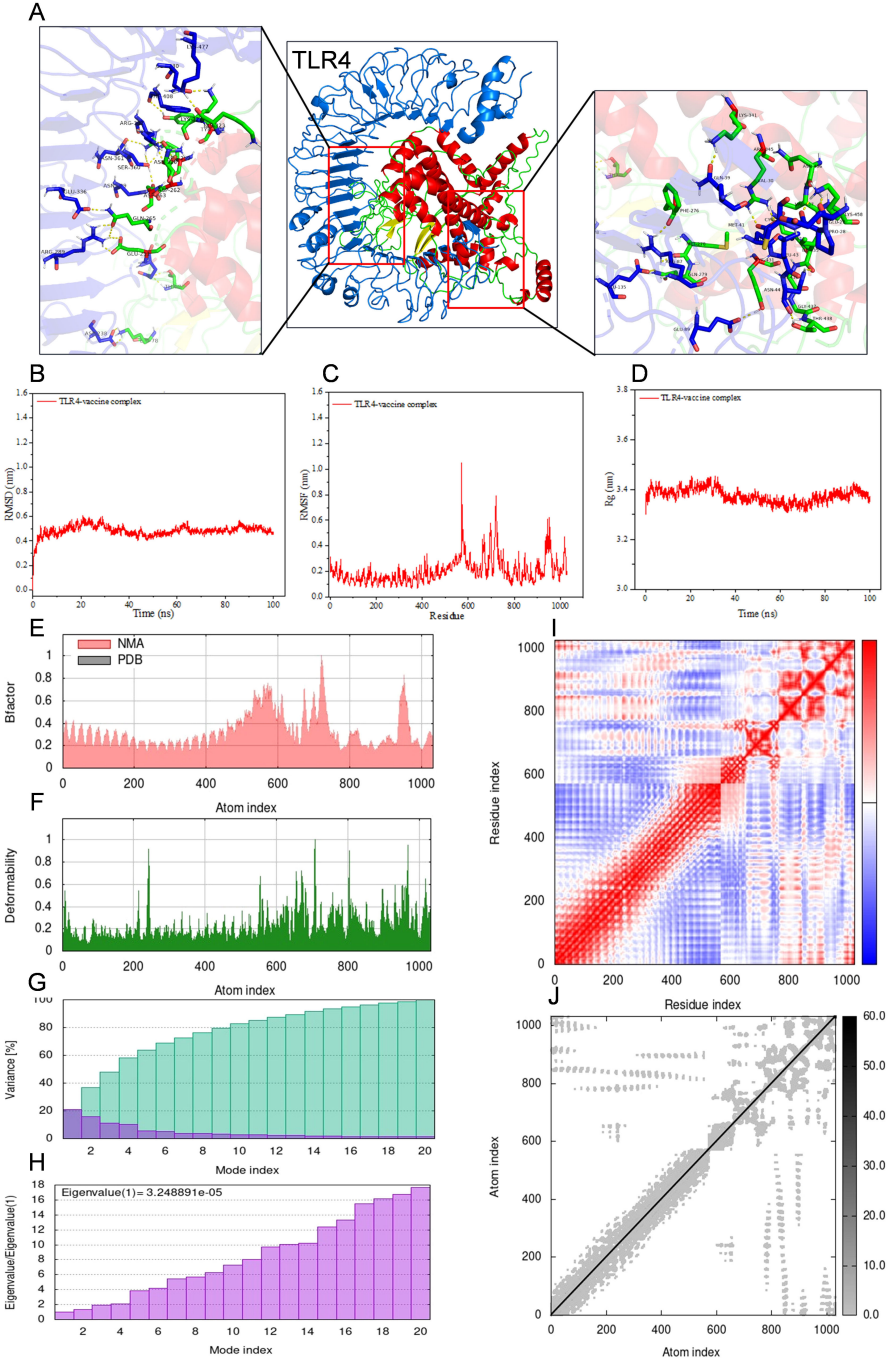
Molecular docking between vaccine and human TLR4 and iMODS results of the docking complex. **(A)** Molecular docking result of vaccine with TLR4. The residues involved in the hydrogen bond are shown on the right-hand side. **(B–D)** Molecular dynamic simulation of the vaccine-TLR4 complex, including RMSD value of the complex backbone, RMSF value of side-chain residues, and radius of gyration during the molecular dynamic simulation. **(E–J)** Results of iMODS of vaccine-TLR4 docking complex. **(E)** B-factor; **(F)** Deformability plot; **(G)** Variance; **(H)** Eigenvalue; **(I)** Covariance matrix analysis; **(J)** Elastic network model.

For the purpose of conducting molecular docking simulations, the refined tertiary structure of the multi-epitope vaccine was subjected to docking with Toll-like receptor 2 (TLR2) and Toll-like receptor 4 (TLR4) utilizing the ClusPro 2.0. Thirty different docked poses were generated for each receptor-ligand complex, exhibiting varied orientations. Given that lower energy scores signify a greater binding affinity, the most favorable docked complex was identified by the lowest energy score. The energy scores recorded for the optimal vaccine-TLR2 and vaccine-TLR4 complexes were -343.9 kcal/mol and -1076.2 kcal/mol, respectively. A representation of the topology and binding interactions of the most stable vaccine-TLR2 complex is depicted in **Figure 5A**, while those of the vaccine-TLR4 complex are illustrated in **Figure 6A**.

Molecular dynamics simulations were conducted on the docked vaccine-TLR complexes using the GROMACS software package. These simulations were executed for a period of 100 nanoseconds (ns) to estimate the dynamic behavior and stability of the complex. The trajectory data generated from the simulations were subsequently analyzed to calculate the root mean square deviation (RMSD) of the protein backbone, the root mean square fluctuation (RMSF) of the side chains, and the radius of gyration (Rg). The graphical representations of the RMSD, RMSF, and Rg values for both the vaccine-TLR2 and vaccine-TLR4 complexes are presented in **Figures 5B–D** and **Figures 6B–D**, respectively, corroborating the relative stability of the docked complexes.

Normal mode analysis utilizing iMODS was employed to investigate molecular motion. The B-factor, deformability profile, variance, eigenvalue, covariance matrix analysis, and elastic network model for both the vaccine-TLR2 and vaccine-TLR4 complexes were elucidated in **Figures 5E–J** and **Figures 6E–J**, respectively. These analyses indicated that the docked complexes exhibited a high degree of stability.

### *In silico* immune simulation for vaccine efficacy

**Figure 7:**
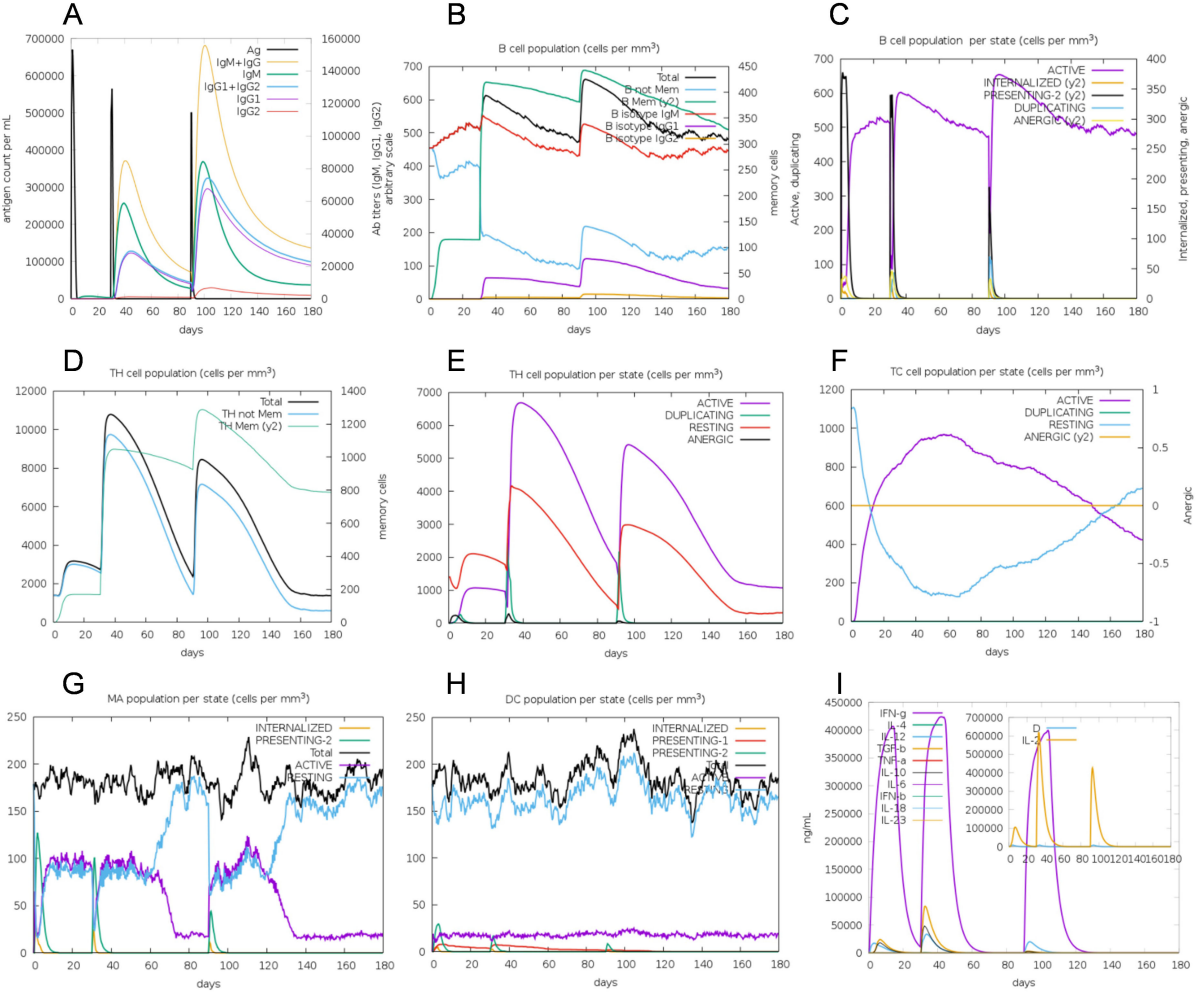
*In silico* simulation of immune response using vaccine as antigen. **(A)** Antigen and immunoglobulins, antibodies are sub-divided per isotype. **(B)** B-cell population. **(C)** B-cell population per state. **(D)** Helper T-cell population. **(E)** Helper T-cell population per state. **(F)** Cytotoxic T-cell population per state. **(G)** Macrophage population per state. **(H)** Dendritic cell population per state. **(I)** Concentration of cytokine and interleukins, D in the inset plot is danger signal.

To assess the immunogenic profile of the multi-epitope vaccine, *in silico* immune simulation was carried out using the C-ImmSim server. The accuracy of this server has been confirmed through both retrospective validation and *in vivo* validation studies (66, 67). The immunoglobulin activity was evident both in secondary and tertiary immune responses (**Figure 7A**). High levels of B-cell, helper T-cell, and cytotoxic T-cell activities were observed during the immune procedure (**Figures 7B–F**). Macrophage activity and dendritic cell activity were also rapidly increased after each exposure (**Figures 7G, H**). High levels of INF-γ, IL-2, and IL-10 were also observed (**Figure 7I**). This immunogenic profile indicates that the multi-epitope vaccine could induce effective immune responses.

### Codon adaptation and *in silico* cloning

**Figure 8:**
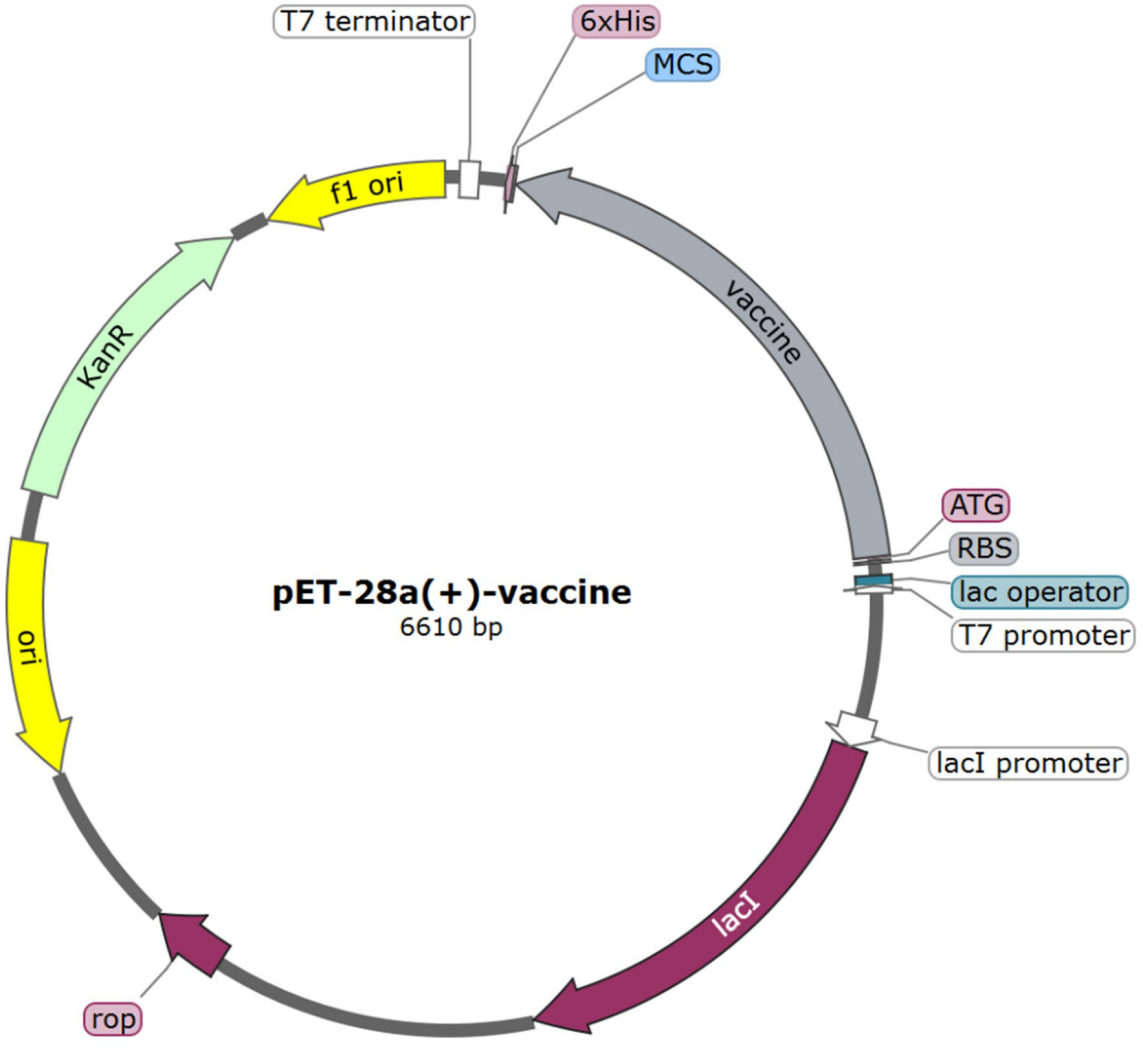
*In silico* cloning of vaccine construct into pET-28a(+) vector. The grey region represents the vaccine encoding gene.

The nucleotide sequence encoding the multi-epitope vaccine was optimized utilizing the online codon optimization tool, ExpOptimizer, with *Escherichia coli* designated as the expression host. Post-optimization, the codon adaptation index (CAI) of the sequence reached 0.8 (with an ideal range of 0.8-1.0), and the GC content was adjusted to 51.82% (within the ideal range of 40%-60%). The 1374-nucleotide sequence was successfully cloned into the pET-28a(+) vector, between the ATG start codon and the XhoI restriction site, using the SnapGene software. The plasmid map of the resulting expression vector is illustrated in **Figure 8**, with the vaccine fragment highlighted in grey.

## Discussion

Vaccines are pivotal in curbing the spread of infectious diseases. Multiepitope vaccines, which harness specific pathogen components to elicit robust immune responses, represent an innovative direction in vaccine development (68, 69). These vaccines are characterized by abbreviated development timelines, reduced costs, and enhanced safety profiles, rendering them exceptionally promising. Current research endeavors are concentrated on the design of *H. pylori* multiepitope vaccines, which have demonstrated efficacy in the prevention and treatment of *H. pylori* infections (70, 71). *In silico* computational vaccine design methodologies offer a more rational and cost-effective approach compared to traditional strategies.

In this study, nine *H. pylori* proteins involved in adhesion, colonization, survival, and virulence were selected for the construction of a multi-epitope vaccine. High-scoring and antigenic epitopes from each protein were chosen as the principal epitopes. To prevent the formation of neo-epitopes, linker sequences were incorporated between the epitopes in the final construct. Secondary structure prediction indicated that the vaccine possessed a flexible and stable conformation conducive to antibody binding. The vaccine was predicted to be non-allergenic, soluble, stable *in vitro*, and thermally stable.

The tertiary structure of the multi-epitope vaccine was predicted, refined, and validated. A high-quality tertiary structure was used for subsequent analyses. Molecular docking revealed that the vaccine could engage with Toll-like receptors (TLR2 and TLR4) with favorable affinity. Molecular dynamics simulations corroborated the high stability of docking complexes. Furthermore, immune simulation based on the vaccine sequence injection suggested that the multi-epitope vaccine exhibited excellent immunogenicity. For experimental evaluation, codon optimization of the multi-epitope vaccine was executed to enhance translational efficiency. The optimized sequence was then cloned *in silico* into the pET-28a(+) plasmid, setting the stage for follow-up experiments.

In summary, the assessment of the *H. pylori* multi-epitope vaccine is encouraging, highlighting its potential applicability and offering novel insights for the advancement of *H. pylori* vaccine development. Future research will require further experimental validation to assess the druggability of the constructed multi-epitope vaccine. Additionally, the strategy employed in this study holds significant potential for the construction of multi-epitope vaccines and should be considered for the development of vaccines against other infectious agents.

## Data Availability

The original contributions presented in the study are included in the article/supplementary material. Further inquiries can be directed to the corresponding authors.
